# Fine-Scale Habitat Associations and Diel Activity Patterns of Camera-Detected Alpine Musk Deer (*Moschus chrysogaster*) in a High-Altitude Tibetan Landscape

**DOI:** 10.3390/ani16162623

**Published:** 2026-08-21

**Authors:** Ziwei Liu, Yuxing Zhan, Yuzhe Huang, Junwen Cao, Guoping Lu, Yongtao Xu, Yuqin Wang, Cheng Huang

**Affiliations:** School of Forestry, Jiangxi Agricultural University, Nanchang 330045, China; liu2820877496@163.com (Z.L.);

**Keywords:** diel activity pattern, habitat association, independent detection event, occupancy model, Qinghai–Tibet Plateau

## Abstract

Fine-scale habitat data for alpine musk deer (*Moschus chrysogaster*) remain scarce across the Qinghai–Tibet Plateau, limiting evidence-based conservation. We deployed 221 camera stations at approximately 10 km grid spacing in Baqing County, Nagqu, Tibet, from August to November 2023, and analyzed 1004 independent detections (30 min threshold) with single-season occupancy models that account for imperfect detection, complemented by logistic and negative binomial models and circular activity analyses. Musk deer were detected at 123 of 221 stations (55.66%). Occupancy was negatively associated with elevation (β = −0.70, 95% CI: −1.02 to −0.38) and positively associated with slope (β = 0.58, 95% CI: 0.24–0.92), while detection probability increased with survey effort; the normalized difference vegetation index (NDVI) and distances to roads, settlements, and water showed weak and unstable support across all frameworks. Activity was broadly cathemeral with a moderate diurnal tendency (Rayleigh test, *p* = 0.001), and activity timing differed among months and between stations near versus far from roads and settlements. These results provide the first occupancy-based evidence of alpine musk deer habitat use on the central Qinghai–Tibet Plateau and identify relatively low-elevation, steep terrain as priorities for conservation planning.

## Simple Summary

The alpine musk deer (*Moschus chrysogaster*) is a globally threatened ungulate inhabiting the high mountains of the Himalaya and the Qinghai–Tibet Plateau, where fine-scale information on its habitat use and activity patterns remains scarce. From August to November 2023, we deployed 221 infrared camera traps across approximately 9810 km^2^ of Baqing County, Tibet, China, and analyzed 1004 independent detections with occupancy models that account for imperfect detection. Musk deer were detected at 55.66% of the stations. Elevation and slope emerged as the strongest correlates of occupancy, with deer favouring relatively lower elevations and steeper terrain. In contrast, vegetation and distance-to-human-feature variables showed weak and inconsistent effects. Activity was broadly cathemeral with a daytime peak, and activity timing differed among months and between stations near versus far from roads and settlements, although these differences require careful interpretation. These findings provide the first occupancy-based insights into alpine musk deer ecology on the central Qinghai–Tibet Plateau and can guide conservation planning for this vulnerable species.

## 1. Introduction

Musk deer (*Moschus* spp.) are small, solitary forest- and alpine-dwelling ungulates endemic to Asia, and most of the seven recognized species face persistent conservation pressure from habitat degradation, illegal hunting for musk, and climate-driven range shifts [1]. The alpine musk deer (*Moschus chrysogaster*) is distributed across two major geographic regions: the southern slopes of the Himalaya (Nepal, northern India including Uttarakhand, Sikkim, and Arunachal Pradesh, and Bhutan) and the Qinghai–Tibet Plateau and its eastern margins in China (Tibet Autonomous Region, Qinghai, Sichuan, Gansu, and northwestern Yunnan) [2,3,4]. Across this range, the species occupies high-elevation grasslands, alpine shrublands, forest-edge habitats, and rugged montane terrain, typically between 3000 and 5000 m above sea level [5,6]. Despite this broad geographic distribution, quantitative data on fine-scale habitat associations and activity rhythms are fragmentary, and systematic camera-trapping studies remain concentrated in a few regions, notably protected areas in Nepal [5,7], Uttarakhand in India [8], and select nature reserves in Sichuan and Qinghai, China [3,9,10].

The ecological requirements of alpine musk deer have been investigated primarily through two complementary approaches: habitat suitability modeling at landscape to regional scales using presence-only or presence-background data [4,11], and site-level analyses of environmental correlates using sign surveys or camera-trap detections [5,7,12]. Landscape-scale studies have consistently identified elevation, terrain ruggedness, and vegetation indices as important predictors of suitable habitat [4,11], while finer-scale work has emphasized the role of shrub cover, slope steepness, and distance to water in shaping local occurrence [5,7]. However, most site-level studies lack the repeated detection histories necessary to estimate and account for imperfect detection—a critical concern for a cryptic, low-density species [13]. Consequently, the extent to which apparent environmental associations reflect true habitat relationships versus variation in detection probability remains uncertain across most of the species’ range.

The Qinghai–Tibet Plateau presents unique challenges and opportunities for understanding alpine musk deer ecology. The region is characterized by extreme elevation, low primary productivity, pronounced seasonality, and widespread pastoral land use [14,15]. In eastern Nagqu Prefecture, where our study was conducted, alpine musk deer coexist with yak and Tibetan sheep grazing, seasonal caterpillar fungus (*Ophiocordyceps sinensis*) collection, and sparse rural settlements distributed along valley bottoms. These multiple land-use pressures may affect musk deer through forage competition, direct disturbance, and altered predation risk, yet their influence on musk deer occurrence and behaviour has rarely been quantified [5,16]. Furthermore, because roads, water systems, and settlements in this mountainous landscape are spatially coupled with terrain position—all concentrated in lower-elevation valleys—disentangling the effects of topography from those of human accessibility and resource distribution requires careful analytical treatment [17].

Diel activity patterns represent another dimension of ecological knowledge that remains poorly documented for alpine musk deer. Existing studies report variable activity rhythms: radio-telemetry work in Sagarmatha National Park, Nepal, suggested predominantly crepuscular activity [2], whereas camera-trap studies in Uttarakhand, India, and Sichuan, China, have reported a higher proportion of daytime records [8,9,18]. In alpine environments, activity timing may be shaped by thermal conditions, predation risk, human disturbance, and seasonal changes in forage availability and energetic demands [19]. However, no published study has examined how alpine musk deer activity patterns vary across months or in relation to human-proximity gradients within the Qinghai–Tibet Plateau.

Here, we address these knowledge gaps using a systematic camera-trapping dataset from 221 stations deployed across the full elevation gradient of Baqing County, Nagqu, Tibet. Our objectives were to: (1) estimate site-level occupancy probability while accounting for imperfect detection, and evaluate environmental correlates of occupancy; (2) assess the robustness of these associations using complementary logistic and count-model frameworks, and test for residual spatial autocorrelation to evaluate our 10 km grid design; and (3) describe diel activity patterns and test whether activity timing differed among months and between stations near versus far from roads and settlements.

Based on the ecology of small mountain ungulates and previous musk deer research, we predicted that occupancy would decline with elevation within the sampled range, reflecting greater resource availability and shorter snow seasons at relatively lower elevations, and would increase with slope steepness, consistent with the topographic refuge hypothesis [20,21]. We further predicted that the normalized difference vegetation index (NDVI) and distance-based proxies would show weaker and less consistent associations than topographic variables, because they capture habitat and disturbance only indirectly, and that activity would be broadly cathemeral with diurnal tendencies, differing between stations near versus far from roads and settlements.

## 2. Materials and Methods

### 2.1. Study Area

**Figure 1:**
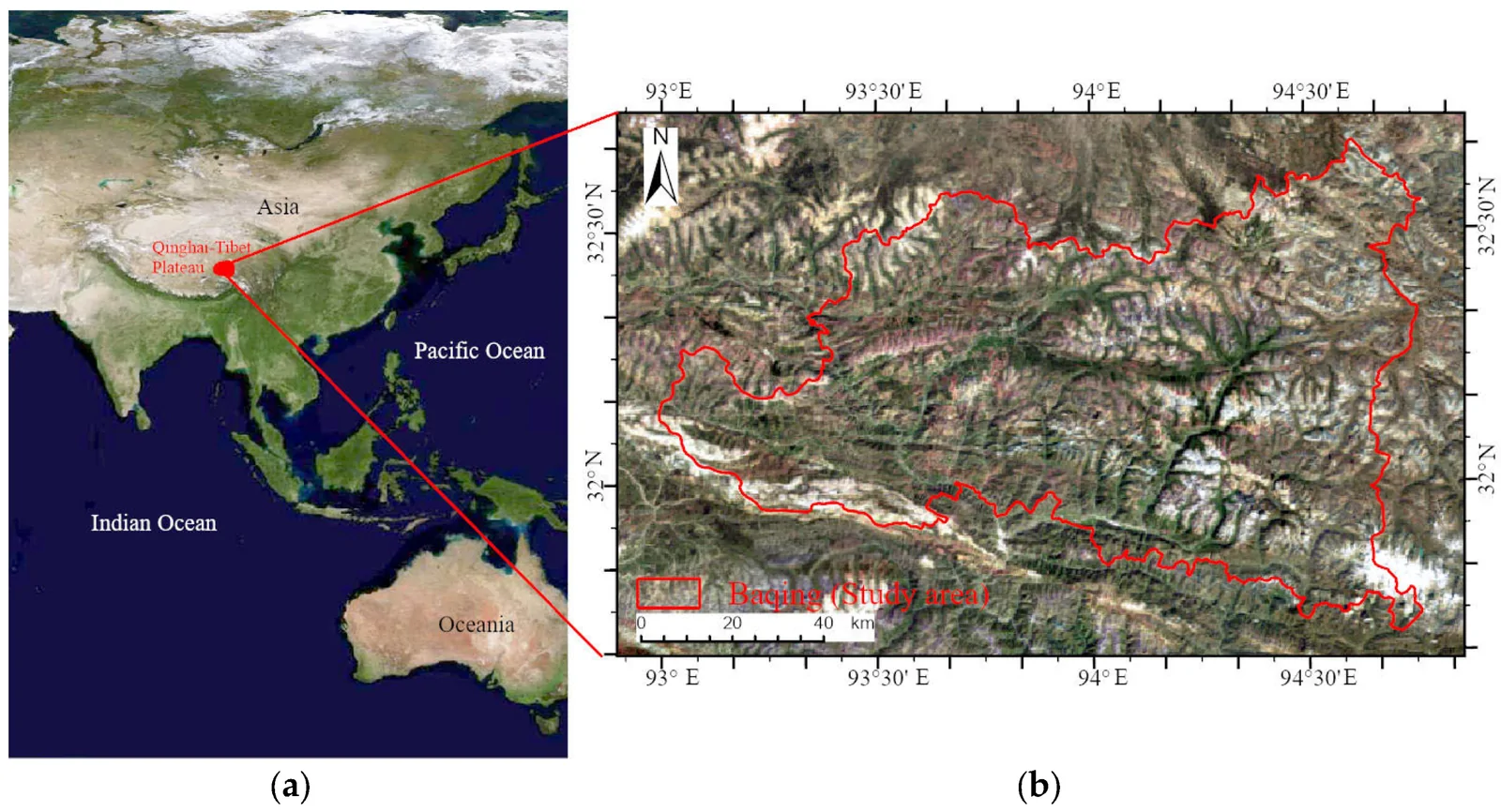
Location of the study area and camera-trap stations in Baqing County, Nagqu City, Tibet Autonomous Region, Asia. (**a**) Location of the study area (red polygon) within Asia; (**b**) Baqing County (red polygon), Tibet, overlaid on the Landsat image; map projection: WGS 1984 UTM Zone 46N.

The study area was located in Baqing County, Nagqu City, Tibet Autonomous Region, China (32°35′ N, 93°55′ E), on the southern slope of the Tanggula Mountains and in the upper reaches of the Nujiang River, representing a typical alpine landscape of the Qinghai–Tibet Plateau (Figure 1). Baqing County covers approximately 9810 km^2^ and has a population density of approximately 5.7 persons/km^2^ according to the Seventh National Population Census in 2020. It is an important pastoral county in eastern Nagqu. The climate is a plateau subfrigid monsoon type, with mean annual temperature of approximately −2 to 0 °C. Mean temperature in the warmest month (July) is approximately 8–10 °C, whereas mean temperature in the coldest month (January) is approximately −12 to −15 °C. Annual precipitation is approximately 450–550 mm, concentrated from June to September [14].

Vegetation is dominated by alpine meadow communities, mainly *Kobresia pygmaea* and *K. humilis*. Alpine shrubs such as *Rhododendron* spp., *Salix* spp., and *Potentilla fruticosa* occur sporadically on sunny slopes and at relatively lower elevations, whereas higher elevations gradually transition to alpine steppe and sparse scree vegetation [22]. The Nujiang River and its tributaries form the main water system, and National Highway G317 crosses the southern part of the county; county and township roads are mainly distributed along valleys. The area lacks a national nature reserve, but Baqing County lies within the Qinghai–Tibet Plateau ecological security barrier and is adjacent to the Lancang River Source Area of Sanjiangyuan National Park in Qinghai.

The local economy is dominated by animal husbandry. Yak and Tibetan sheep grazing is widespread across grasslands at elevations of approximately 3800–5000 m. Grazing may influence habitat use by wild ungulates through forage competition, trampling disturbance, and indirect changes in predation risk [5]. Caterpillar fungus (*Ophiocordyceps sinensis*) collection from May to July is another important economic activity and may increase seasonal human presence in alpine meadows. The study area contains no industrial facilities, and settlements are sparsely distributed along valleys.

**Figure 2:**
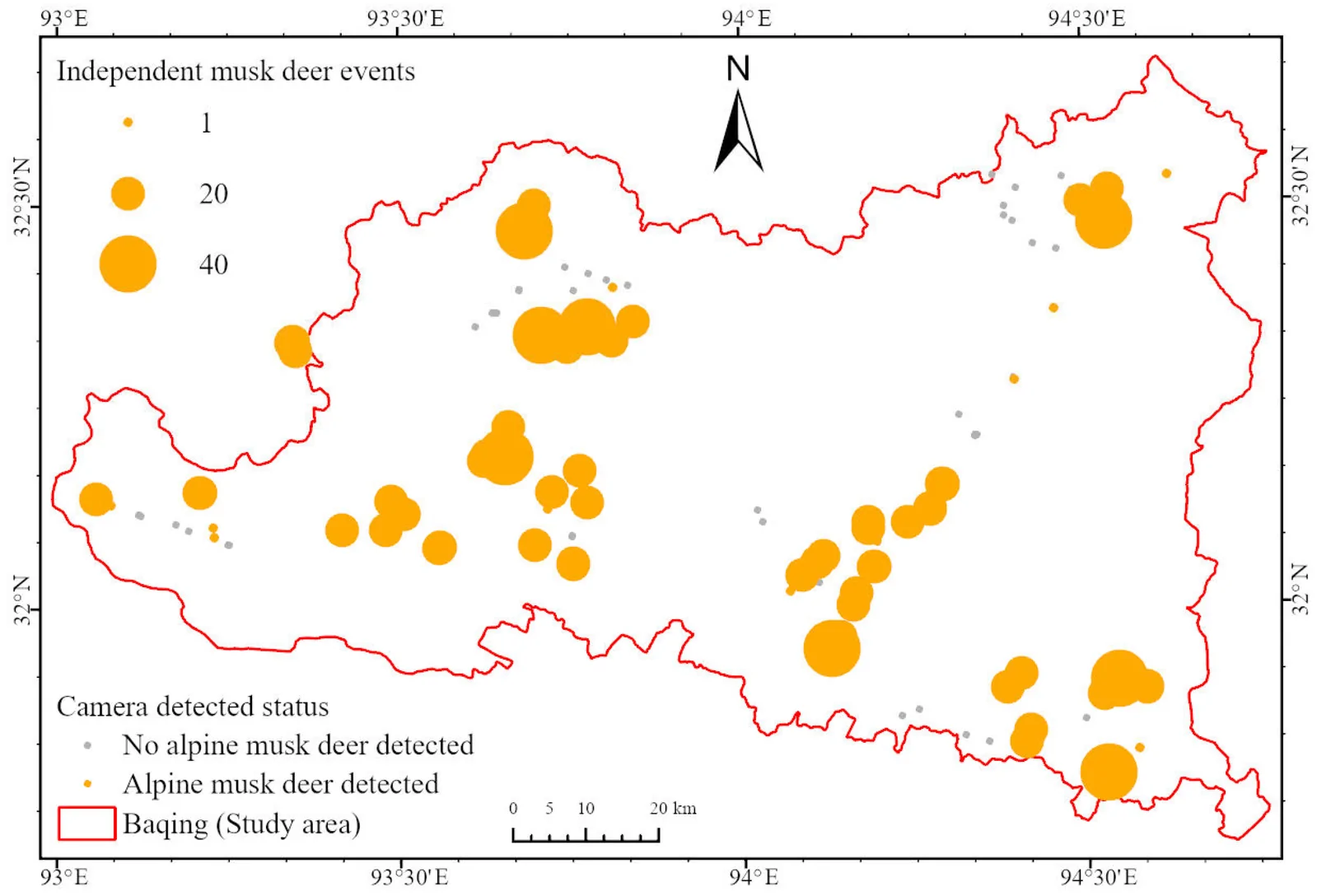
Camera stations in the study area and stations with camera-detected Alpine musk deer.

**Figure 3:**
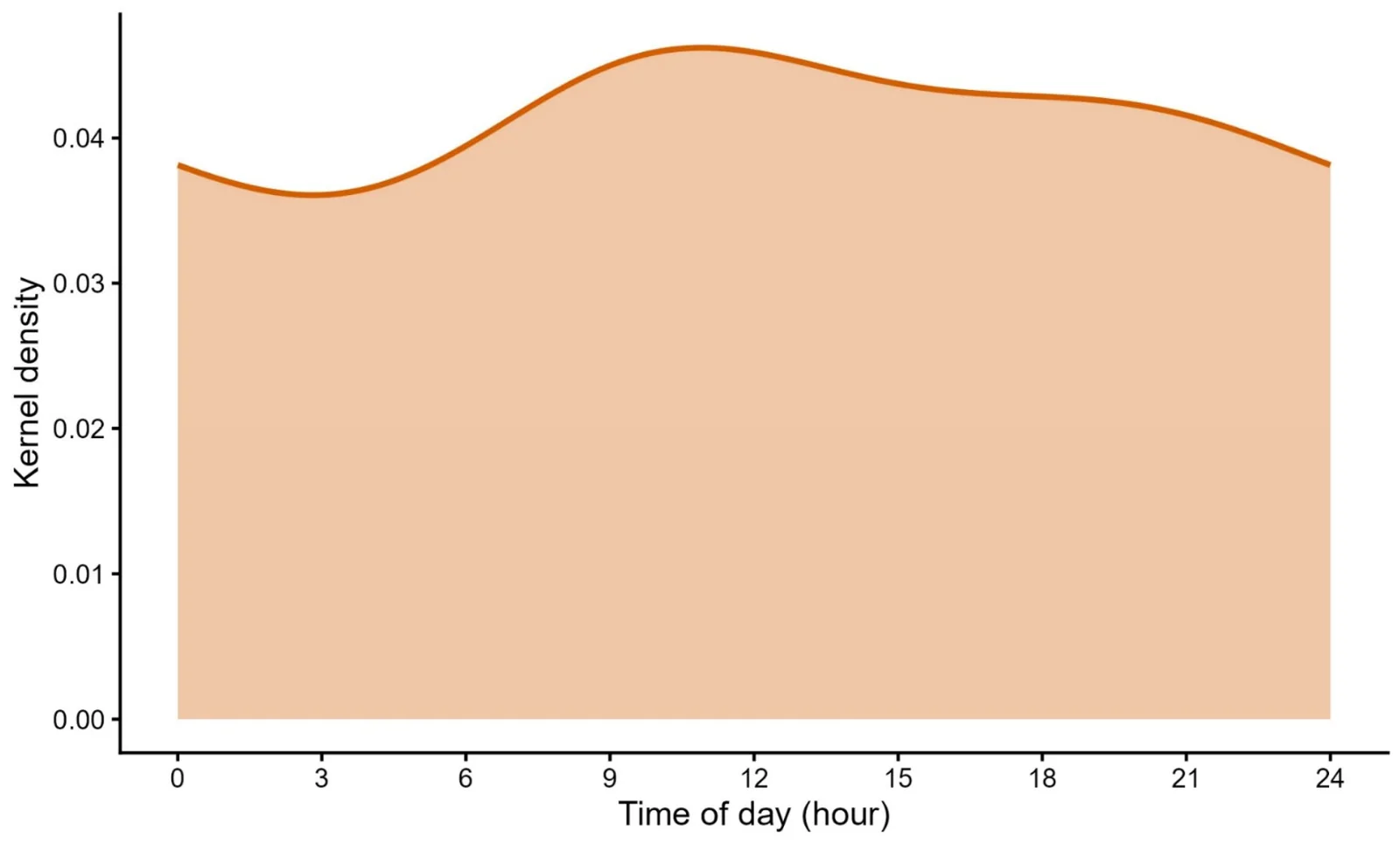
Diel activity density curve of Alpine musk deer based on 30 min independent events.

**Figure 4:**
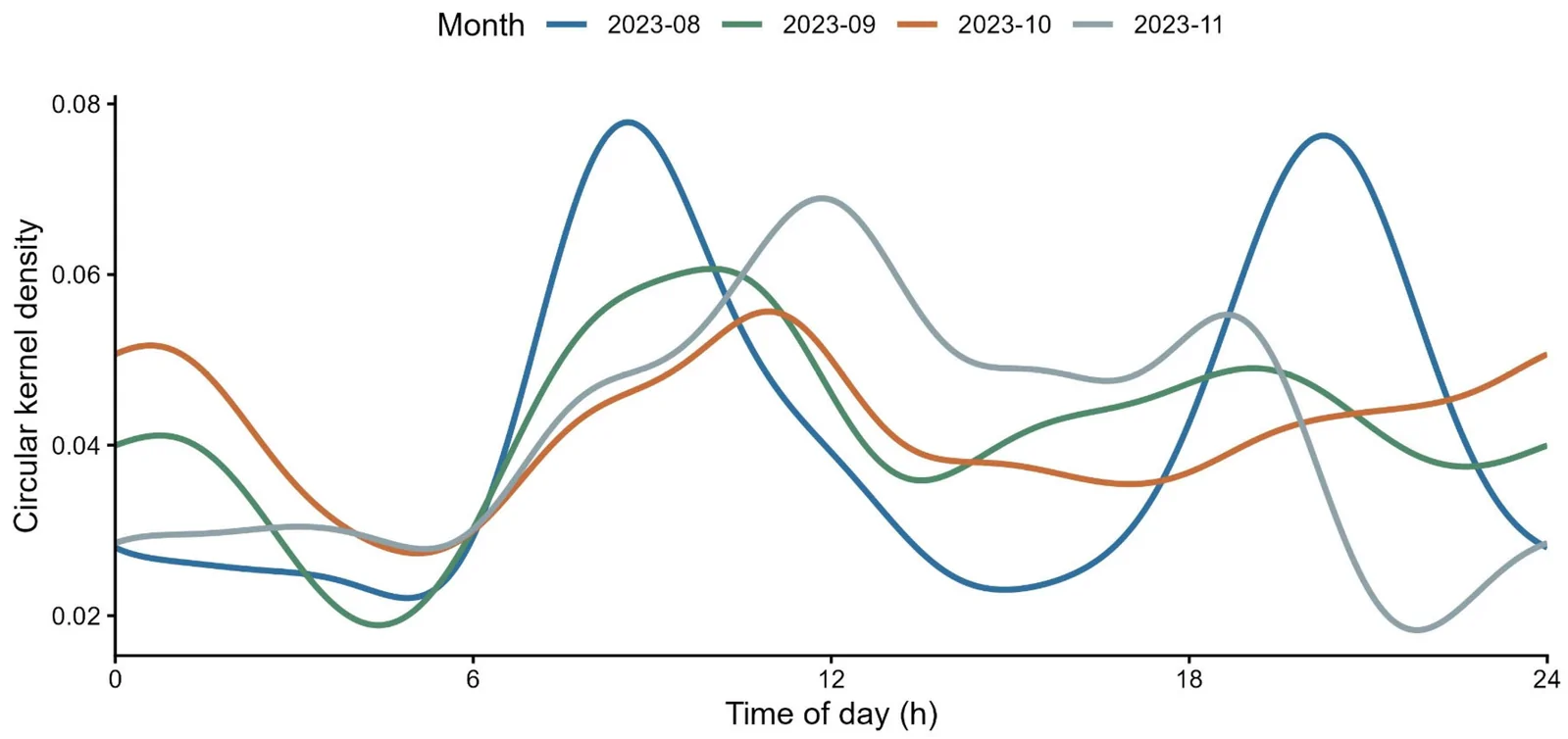
Holm-corrected monthly diel activity curves of camera-detected alpine musk deer.

In this study, Figure 1 and Figure 2 were generated with QGIS 3.44, Figure 3 and Figure 4 were drawn using Python 3.11, and all data analyses and model building were performed in Python 3.11.

### 2.2. Camera-Trap Deployment and Data Processing

Beginning in August 2023, 221 infrared camera traps (Loreda passive infrared camera, Loreda Technology Co., Shenzhen, China) were deployed for wildlife monitoring. For each camera station, camera ID, geographic coordinates, installation date, retrieval date, and total monitoring days were recorded. Mean monitoring duration per station was 85 days (median 93, range 6–275 days). Stations that operated for fewer than 14 days (*n* = 8) were retained in occupancy analyses because the multi-occasion framework accommodates variable sampling durations; sensitivity analyses excluding these stations produced qualitatively identical results.

Camera traps were deployed using a systematic grid-based design, with approximately 10 km between neighboring stations. This spacing was chosen to cover the full elevation gradient of the study area (approximately 4142–5122 m) and the associated variation in slope, aspect, and vegetation [23,24]. At this spacing, stations were treated as independent sampling units; the adequacy of this assumption was evaluated with the residual spatial-autocorrelation analyses described in Section 2.5. Stations were not deliberately placed along animal trails or at sites showing signs of animal activity. Instead, coordinates were generated systematically, and in the field, suitable installation locations were selected within approximately 50 m of each preset coordinate. Selection criteria at this micro-scale included: a stable mounting substrate (rock face or sturdy shrub stem), camera orientation parallel to the slope or toward potential animal passage directions (but not deliberately aimed at animal trails), and avoidance of direct exposure to rising or setting sun. This protocol reduces the potential inflation of detection probability associated with preferential placement on animal trails [25,26].

One infrared-triggered passive camera was installed at each station, mounted approximately 40–50 cm above the ground. Cameras were set to photo mode, with passive infrared motion triggering. After triggering, cameras took two to three consecutive photographs, and the trigger interval was set to 0 s. No bait or attractant was used. Camera-trap event records included camera ID, capture date, capture time, species name, and station metadata. Species names were standardized following the Handbook of the Mammals of China [27].

To define independent events, a 30 min threshold was applied as the primary criterion: consecutive records of the same species at the same station were treated as a new independent event only if separated by at least 30 min [28]. Independent event counts were also calculated using a 60 min threshold for sensitivity analysis; results were highly consistent (968 vs. 1004 events), and all main analyses used the 30 min threshold. A station was classified as “detected” if it recorded at least one independent alpine musk deer event. For each detected station, we calculated the number of independent events and the relative abundance index (RAI), defined as the number of independent events per 100 camera-days [29].

### 2.3. Environmental Variables

Environmental variables included elevation (extracted from the Shuttle Radar Topography Mission 1-arcsecond digital elevation model (DEM), approximately 30 m resolution; hereafter “elevation”), slope and aspect (derived from DEM), land-use type (ESRI 10 m resolution), NDVI (median growing-season composite of Landsat 8/9 OLI imagery), and Euclidean distances to roads (DisRoad), settlements (DisJM), and water bodies (DisWater; Table 1). Aspect was transformed into northness (cos(aspect)) and eastness (sin(aspect)) components to preserve circular information [30]. Distance variables were calculated from vector layers: roads were manually digitized from Google Earth high-resolution imagery, settlement points were identified from the same imagery and verified against OpenStreetMap data, and water bodies were extracted from the ESRI land-use layer supplemented by manual digitization.

Before modeling, all continuous environmental variables were standardized (mean = 0, SD = 1) to facilitate comparison of effect sizes. Survey effort was represented by total camera-trap days (log-transformed and standardized for logistic models; log-transformed as an offset for count models). For occupancy models, survey effort was represented by the number of active days within each occasion, included as a detection covariate.

Continuous environmental variables were weakly to moderately correlated (all |r| < 0.30 except DisRoad–DisWater, r = 0.74; Figure S1), and variance inflation factors were low (Elevation = 1.22, Slope = 1.08, NDVI = 1.12, DisRoad = 2.33, DisWater = 2.29, DisJM = 1.08), indicating no severe multicollinearity. The moderate DisRoad–DisWater correlation was considered when interpreting models containing both variables.

### 2.4. Occupancy Modeling

We fitted single-season occupancy models using the unmarked package [31] in R version 4.3. Detection histories were constructed by dividing the monitoring period into 27 occasions of 10 days each (occasion length selected to balance the number of replicates against the need for sufficient detection events per occasion). A station was coded as 1 for an occasion if at least one independent alpine musk deer event occurred during that 10-day window, and 0 otherwise. Stations operating for fewer than 10 days within a given occasion (e.g., because of late deployment or early retrieval) were assigned NA for that occasion, which unmarked handles via the standard missing-data mechanism.

Occupancy probability (ψ) was modeled as a function of environmental covariates: elevation, slope, NDVI, and distance variables (DisRoad, DisJM, DisWater). Detection probability (p) was modeled as a function of survey effort (active camera-days within the occasion, log-transformed and centered) and occasion number (to account for potential temporal trends in detection). We fitted a candidate set of models representing alternative hypotheses about occupancy (Table 2) and compared models using Akaike’s Information Criterion (AIC) [32]. We used the best-supported model for inference, and generated model-averaged predictions when multiple models received substantial support (ΔAIC < 2).

### 2.5. Complementary Models and Spatial Diagnostics

As complementary analytical frameworks, we first fitted binomial logistic generalized linear models (GLMs) to site-level detection status (detected vs. not detected) in relation to the environmental covariates, with log-transformed survey effort as a covariate. We compared four candidate models paralleling the occupancy candidate set using AIC (Table S1), and converted coefficients to odds ratios (OR). Second, to analyze variation in independent event counts among stations, we fitted Poisson, negative binomial, and zero-inflated negative binomial (ZINB) GLMs with log-transformed monitoring days as an offset. We evaluated overdispersion in the Poisson model using the Pearson residual dispersion parameter, and we compared models using AIC; results are presented as incidence rate ratios (IRR). We emphasize that independent event counts index detection intensity, not abundance, because individual identification was not possible and the same animal may trigger the same camera multiple times [33]. Third, to evaluate whether the 10 km grid adequately captured spatial structure, Moran’s I was computed on the Pearson residuals of the best-supported logistic and count models using symmetrized k-nearest-neighbour spatial weight matrices (k = 4, 6, and 8; ape package). As a complementary approach, we fitted spatial-block generalized linear mixed models (GLMMs) with 10 k-means spatial blocks derived from station coordinates as a random intercept (lme4, glmmTMB) and compared AIC with the non-spatial counterparts.

### 2.6. Diel Activity Analysis

Capture times of independent alpine musk deer events were converted to decimal hours and then to circular angles. Diel activity was visualized with a wrapped Gaussian kernel density estimator following Ridout and Linkie [34]; bandwidth was selected using the default method in the overlap package, and a fixed bandwidth of 1.2 h was used for the monthly and human-proximity curves to smooth short-term camera-event noise. The Rayleigh test was applied to evaluate departures from a uniform circular distribution [35]; because this test is most sensitive to unimodal departures, results were interpreted together with the kernel density curves. Monthly variation in activity timing (August–November) was assessed by comparing monthly circular time distributions using the Mardia–Watson–Wheeler (MWW) non-parametric test [36], with pairwise comparisons adjusted for multiple testing using Holm’s correction and validated by bootstrap resampling (200 iterations). To explore potential anthropogenic influences on activity timing, stations were classified as “near” or “far” from anthropogenic features using the site-level median of the minimum distance to either roads or settlements (threshold = 480.9 m); Diel activity curves were plotted for each group and compared with the MWW test. We emphasize that this classification is a crude spatial proxy and does not measure traffic volume, human activity intensity, or livestock presence [17]. Finally, as a descriptive analysis, temporal overlap between alpine musk deer, red fox (*Vulpes vulpes*), and Tibetan fox (*V. ferrilata*) was computed using the coefficient of overlap (Δ) from the overlap package; because this analysis does not incorporate spatial co-occurrence or dietary data, it is reported in the Supplementary Materials (Figure S2). These circular-activity and overlap procedures follow those applied in recent camera-trap studies of mammals [37].

## 3. Results

### 3.1. Sampling Effort and Detection Records

Cameras operated for a mean of 85 days per station (median 93, range 6–275), and the full environmental dataset for the 221 stations had no missing values for any continuous variable (Table S2). The event dataset contained 2128 wildlife detection records, of which alpine musk deer contributed 1004 independent events under the 30 min threshold (968 under the 60 min threshold); red fox (944 events) and Tibetan fox (179 events) were the next most frequently recorded species. Alpine musk deer were detected at 123 of 221 stations (55.66%), with a mean of 4.5 independent events per station (median 1, range 0–59; Figure 2), corresponding to a mean recording intensity of 5.7 events per 100 camera-days. Station land use was dominated by grassland (211 stations), with nine shrubland and one forest station; all shrubland and forest stations recorded musk deer, but these sample sizes were too small for formal inference.

### 3.2. Occupancy and Environmental Correlates

Across the 221 stations, mean elevation was 4535 m (range 4142–5122 m) and mean slope was 20° (range 1–52°); median distances to roads, water, and settlements were 481 m, 1092 m, and 13,942 m, respectively. In univariate comparisons, stations with detections were at lower elevations (median 4470 vs. 4599 m), on steeper slopes (median 22° vs. 16°), and closer to roads (median 360 vs. 742 m) and water (median 700 vs. 1437 m) than stations without detections (Wilcoxon tests, all *p* ≤ 0.03; Table S3, Figure S3), whereas NDVI, distance to settlements, aspect, and monitoring duration did not differ between groups (all *p* > 0.05). These univariate patterns were confirmed in the multivariate occupancy framework below.

The best-supported occupancy model included elevation and slope as covariates of occupancy, with survey effort and occasion as covariates of detection (Occu 2; Table 3). The topography + vegetation model (Occu 3, ΔAIC = 2.02) received modest support, whereas the null model (ΔAIC = 46.82) and models adding NDVI, DisRoad, DisJM, or DisWater (ΔAIC ≥ 4.80) received substantially less support. In the best-supported model (Table 4), occupancy probability was negatively associated with elevation (β = −0.70, SE = 0.16, *p* < 0.001; OR = 0.50, 95% CI: 0.36–0.68) and positively associated with slope (β = 0.58, SE = 0.17, *p* < 0.001; OR = 1.79, 95% CI: 1.27–2.51). Predicted occupancy declined from approximately 0.83 near the minimum sampled elevation (~4150 m) to 0.16 near the maximum (~5120 m) and increased from approximately 0.33 on gentle terrain to 0.87 on the steepest slopes (52°; Figure S4). Detection probability increased with survey effort (β = 0.15, SE = 0.05, *p* = 0.005) and varied modestly among occasions (0.11–0.18; mean 0.14), consistent with moderate detectability of a cryptic ungulate.

### 3.3. Robustness Across Analytical Frameworks and Spatial Independence

Among the logistic GLMs (Tables S4 and S5), the topography-vegetation-effort model had the lowest AIC (277.98), followed by the water-settlement (ΔAIC = 2.13) and road-settlement (ΔAIC = 3.57) models. In the best-supported logistic model (Table S6), detection odds decreased with elevation (OR = 0.48, 95% CI: 0.35–0.67) and increased with slope (OR = 1.82, 95% CI: 1.31–2.52), while NDVI (OR = 0.97), DisRoad (OR = 1.08), and DisJM (OR = 0.93) showed no significant associations. Estimates were highly consistent between the occupancy and logistic frameworks (Table S6), although logistic ORs were modestly inflated relative to occupancy-derived ORs, likely reflecting unmodeled detection heterogeneity.

The Poisson count model showed extreme overdispersion (Pearson dispersion parameter = 14.07, AIC = 2486.9); the negative binomial model fitted substantially better (AIC = 1039.0; Table S7) and the ZINB model marginally worse (AIC = 1040.9). In the negative binomial model (Table 5), slope was positively associated with detection intensity (IRR = 1.41, 95% CI: 1.08–1.86), elevation showed a negative but non-significant trend (IRR = 0.78, 95% CI: 0.60–1.03), and NDVI, DisRoad, and DisJM showed no significant associations.

Residual spatial autocorrelation was weak in magnitude. Moran’s I on the Pearson residuals of the logistic model was positive but modest (I = 0.12–0.15, *p* < 0.01 at k = 4, 6, and 8), and the negative binomial residuals showed weaker, inconsistent autocorrelation (significant only at k = 6, *p* = 0.022; Table S8). Spatial-block GLMMs (10 k-means spatial blocks as a random intercept) produced AIC values nearly identical to the non-spatial models (logistic: 278.1 vs. 278.0; negative binomial: 1041.2 vs. 1039.0), indicating that the spatial dependence was weak and did not materially affect inference; we therefore report the non-spatial models and discuss this limitation in Section 4.3.

### 3.4. Diel Activity Patterns and Their Variation

Independent events were recorded between 15 August and 25 November 2023, with a mean recorded time of 12:36 h (median 11:49 h). Events occurred throughout the 24-h cycle, with higher frequencies between 07:00 and 12:00 h and a secondary peak between 18:00 and 20:00 h (Figure 3). The kernel density curve was broad rather than narrowly unimodal; the Rayleigh test rejected uniformity (test statistic = 0.082, *p* = 0.001), indicating weak concentration of activity around midday. We therefore describe the pattern as cathemeral with a moderate diurnal tendency.

Monthly event counts ranged from 167 (November) to 338 (September), with standardized recording intensity of 5.08–6.27 events per 100 camera-days (Table S9). Monthly activity curves had broadly similar shapes, but the MWW global test indicated significant heterogeneity (W = 12, *p* < 0.001; Figure 4). Pairwise tests with Holm correction showed that August differed from November (W = 10, adjusted *p* = 0.004) and October differed from November (W = 9, adjusted *p* = 0.009); other comparisons were not significant (Figure S5). Bootstrap validation (200 iterations) confirmed these patterns (Table S10). Because November had substantially fewer effective camera-days (2664 vs. 6359 in September), the apparent November shift may partly reflect reduced sampling coverage rather than a true seasonal change; these comparisons are restricted to August–November and cannot be extrapolated to other seasons.

Diel activity curves differed between stations near anthropogenic features (minimum distance to road or settlement ≤ 480.9 m; *n* = 644 events) and stations far from them (*n* = 360 events; MWW test, W = 22, *p* < 0.001; Figure S6): the near group showed slightly higher nighttime and early-morning activity, whereas the far group showed more concentrated daytime activity. Bootstrap validation supported this difference (all *p* < 0.001). Because the near/far classification is a crude spatial proxy that does not measure traffic volume, human activity intensity, or livestock presence, these results should not be interpreted as evidence of a direct behavioral response to human disturbance.

## 4. Discussion

This study provides the first occupancy-based analysis of environmental correlates of alpine musk deer occurrence in the central Qinghai–Tibet. From 1004 independent detections at 123 of 221 stations, elevation and slope emerged as the most robust correlates of site-level occupancy: occupancy probability declined with elevation and increased with slope, whereas NDVI and distances to roads, settlements, and water showed weak and unstable support across occupancy, logistic, and count models. Diel activity was broadly cathemeral with a diurnal tendency, and activity timing differed among months and between stations near versus far from roads and settlements, although these differences require cautious interpretation. Residual spatial autocorrelation was weak in magnitude and did not materially affect inference, supporting the adequacy of our 10 km grid design for this species and landscape.

### 4.1. Topographic Gradients as the Dominant Environmental Correlates

The negative association between occupancy probability and elevation within the sampled range (4142–5122 m) is consistent with our prediction and with a broader body of evidence suggesting that elevation functions as a master environmental filter in high-altitude ecosystems [20,38]. Within our study area, even relatively modest elevation differences of a few hundred meters correspond to measurable changes in temperature, snow-cover duration, and growing-season length [14]. Relatively lower elevations within this high-altitude landscape may offer greater vegetation productivity, reduced energetic costs of thermoregulation and locomotion in snow, and longer snow-free periods [39]. These findings are broadly consistent with occupancy or habitat-use studies of other alpine ungulates on the Qinghai–Tibet Plateau, including Tibetan gazelle (*Procapra picticaudata*) [40], blue sheep (*Pseudois nayaur*) [41], and white-lipped deer (*Przewalskium albirostris*) [42], each of which shows a tendency to use lower portions of their respective elevation gradients. However, the specific elevation zones and the slope of the relationship differ among species, reflecting taxon-specific physiological tolerances, dietary requirements, and predator-avoidance strategies.

The positive association between slope and occupancy probability aligns with the topographic refuge hypothesis [21,43]. Steep, complex terrain may benefit small ungulates through three non-mutually-exclusive mechanisms: (1) reduced accessibility for large predators and humans or livestock, creating de facto refuge areas; (2) increased availability of microtopographic features that provide concealment and escape options; and (3) locally reduced snow accumulation on windward slopes or through gravitational redistribution [22]. Our finding that slope was the only variable positively associated with both occupancy probability and detection intensity (count models) suggests that steeper stations not only had a higher probability of occupancy but also supported more frequent camera-triggering events—potentially reflecting higher local use intensity or repeated passage of resident individuals along terrain-constrained movement corridors.

These topographic associations have been reported in other musk deer populations. In Nepal, Khadka and James [5] found that Himalayan musk deer (*M. chrysogaster*, treated by some authorities as conspecific with alpine musk deer) selected steeper slopes and avoided livestock-occupied areas. In Uttarakhand, India, Syed and Ilyas [8] reported that alpine musk deer used rugged terrain with dense understory vegetation. Camera-trap studies of alpine musk deer in the Helan Mountains of northern China [44] and forest musk deer (*M. berezovskii*) in Sichuan [9,18] have also identified elevation and terrain ruggedness as important habitat predictors. The consistency of these topographic associations across widely separated geographic populations—spanning the southern Himalaya, the eastern Qinghai–Tibet Plateau, and the arid mountains of northern China—suggests that the elevation–slope combination represents a fundamental habitat template for *Moschus* species, within which vegetation composition, disturbance regime, and other local factors modulate habitat suitability.

### 4.2. Limited Explanatory Power of Vegetation and Distance-Based Proxies

NDVI showed no significant association with occupancy, detection, or detection intensity in any of our models. This null result is informative and has at least two plausible explanations. First, NDVI measured at 30-m resolution from Landsat imagery is a coarse indicator of overall vegetation greenness that may not capture the fine-scale vegetation structure—such as shrub patch density, forb species composition, or forage quality—that is relevant to a small, selective browser like the alpine musk deer [45]. Second, the study area is dominated by relatively homogeneous alpine meadow, and the range of NDVI values (0.08–0.52) may have been insufficient to detect a signal against the stronger topographic gradient. Future studies incorporating field-measured variables such as shrub cover, forage plant diversity, or biomass would better resolve vegetation–occurrence relationships [5,46].

The weak and unstable associations for DisRoad, DisJM, and DisWater across models reflect the spatial coupling of these variables with terrain in alpine valley landscapes. Roads in the study area predominantly follow river valleys, which simultaneously represent lower elevations, greater water availability, and easier passage corridors. Consequently, DisRoad simultaneously carries information about terrain position, resource availability, and human accessibility [17]. The moderate correlation between DisRoad and DisWater (r = 0.74) further confirms this spatial entanglement. The univariate finding that detected stations were closer to roads should not be interpreted as evidence that alpine musk deer prefer roads or are unaffected by them; rather, it likely reflects the co-occurrence of roads and favorable topographic conditions in valley bottoms. DisJM faces a parallel interpretive challenge: settlements in Baqing County are small and sparsely distributed (the county’s population density of approximately 5.7 persons/km^2^ is among the lowest in China; cf. national average of approximately 147 persons/km^2^), and settlement locations are themselves constrained by topography, occurring almost exclusively in broad valley bottoms.

These interpretive limitations highlight a broader inferential challenge in wildlife–habitat studies that rely solely on remote-sensing proxies for human disturbance [47]. Our anthropogenic-proximity activity comparison—which showed significant differences in diel activity curves between stations near versus far from roads and settlements—provides a suggestive signal that human-associated features may influence musk deer behavior. However, without direct measures of traffic volume, grazing intensity, or human presence, we cannot distinguish among the effects of roads as disturbance corridors, roads as terrain features, and roads as indicators of other spatially structured processes. We strongly recommend that future studies in this system incorporate field-based measures of grazing intensity (e.g., yak and sheep density transects), seasonal human presence (e.g., caterpillar fungus collection camp locations and sizes), and road-use intensity (e.g., traffic counts by vehicle type), ideally within an occupancy or N-mixture framework that explicitly separates habitat use from detection probability [13,48].

### 4.3. Consistency Across Analytical Frameworks and Spatial Independence

The close qualitative agreement between occupancy, logistic, and count models indicates that detection heterogeneity—although present (mean per-occasion *p* = 0.14, increasing with survey effort)—was not severe enough to qualitatively alter ecological inference in this dataset; nevertheless, we recommend occupancy or N-mixture designs as standard practice [33,48]. Residual spatial autocorrelation was weak (Moran’s I = 0.12–0.15 for the logistic model; Table S8), and spatial-block GLMMs did not improve model fit, indicating that the 10 km grid captured most of the spatial structure at the analytical grain of our models. The strong overdispersion in event counts (dispersion parameter = 14.07) is typical of camera-trap data [49] and confirms that independent event counts and RAI should be interpreted as detection intensity rather than abundance.

### 4.4. Diel Activity Patterns in Comparative Context

The cathemeral activity pattern with a diurnal tendency observed in our study is broadly consistent with, but distinct in detail from, activity patterns reported for *Moschus* species elsewhere. Green [2] described Himalayan musk deer in Sagarmatha National Park, Nepal, as predominantly crepuscular, based on radio-telemetry observations. Camera-trap studies in Uttarakhand, India, reported a higher proportion of daytime activity [8], closer to our observations. Forest musk deer in Sichuan showed broadly cathemeral activity with slightly elevated daytime records [9,18], while Siberian musk deer (*M. moschiferus*) in the Russian Far East exhibited stronger diurnal activity during winter, possibly to reduce thermoregulatory costs [50].

These inter-study differences likely reflect genuine variation in activity rhythms driven by latitude, season, predation risk, human disturbance regime, and methodological differences between radio-telemetry and camera-trapping [49]. Our study was restricted to August–November, spanning the late growing season and autumn transition. During this period, alpine musk deer may increase daytime foraging activity to accumulate body reserves for winter [2]. The absence of data from the winter snow season (December–March) and spring breeding season (April–May) is a significant limitation, and our results cannot be extrapolated to annual activity patterns.

The significant monthly variation in activity timing—particularly between August and November and between October and November—should be interpreted cautiously. Although MWW tests detected statistical differences, these could reflect reduced sample sizes in November (167 events from 2664 camera-days) and potentially changing detection conditions (e.g., snow cover affecting camera triggering) rather than genuine behavioral shifts. Year-round monitoring with consistent sampling effort across seasons is needed to resolve seasonal activity patterns.

The significant difference in activity curves between stations near versus far from roads and settlements is provocative but requires follow-up investigation. If alpine musk deer adjust their activity timing in response to diel patterns of human activity (e.g., shifting toward more nocturnal behavior near roads), this could represent a behavioral mechanism for persisting in human-modified landscapes [51]. However, our near/far classification is a crude spatial proxy, and the observed pattern could also reflect habitat differences between valley-bottom stations (near roads) and upland stations (far from roads) that are unrelated to human activity per se.

### 4.5. Conservation Implications

Our findings have several implications for alpine musk deer conservation in the Qinghai–Tibet Plateau. First, the consistency of topographic associations—lower elevations and steeper slopes within the high-altitude landscape—provides a simple, mappable basis for identifying areas likely to support musk deer in Baqing County and potentially in similar landscapes across eastern Nagqu. Conservation planning can prioritize the integrity of steep, rugged hillslopes and the valley-edge transition zones that combine relatively lower elevation with complex terrain.

Second, the weak performance of remote-sensing proxy variables for human disturbance underscores the need for direct, field-based measures of anthropogenic pressure in future research and monitoring. Conservation management decisions that rely solely on distance-to-road or distance-to-settlement metrics risk conflating terrain effects with disturbance effects and may misidentify the actual drivers of musk deer distribution. Incorporating grazing-intensity surveys, seasonal human presence data, and road-use monitoring into conservation planning would substantially strengthen the evidence base [5,17].

Third, the seasonal limitation of our monitoring window (August–November) means that critical information on winter habitat use—when snow cover may restrict movements and concentrate animals in favorable microhabitats—is lacking. Winter is likely a period of elevated mortality risk for alpine ungulates [39], and identifying winter-range characteristics should be a priority for future research. Continuous year-round camera-trapping, combined with seasonal habitat surveys and, ideally, GPS-tracking of individual musk deer, would address this gap.

Finally, our occupancy-modeling framework provides a template for long-term monitoring. By establishing a baseline estimate of occupancy probability against which future surveys can be compared, and by demonstrating that detection probability can be estimated from the existing sampling design, this study lays the groundwork for a monitoring program capable of detecting population trends—a critical need for a species listed as Endangered on the IUCN Red List [52] and as a Class I nationally protected species in China.

### 4.6. Limitations

Several limitations of this study should be acknowledged. First, our monitoring period was restricted to August–November 2023 for alpine musk deer detections (three stations continued monitoring through May 2024 but recorded no further musk deer events, precluding seasonal analysis). All results—particularly occupancy estimates and activity patterns—apply specifically to the late-growing-season and autumn transition period and should not be interpreted as annual averages.

Second, although our occupancy models explicitly estimate detection probability, the assumption of population closure within the 270-day sampling season (27 occasions of 10 days) may be violated if individuals moved on and off sites. For a relatively sedentary, solitary species such as alpine musk deer, this assumption is likely reasonable at the 10 km grain of our sampling grid, but formal testing would require individual identification.

Third, our environmental variables are all derived from remote sensing and GIS layers and do not include direct field measurements of vegetation structure, forage availability, livestock density, or human activity. As discussed above (Section 4.3), this limits the ecological resolution of our inferences, particularly with respect to human-disturbance effects.

Fourth, individual identification was not possible from camera-trap images, and independent event counts cannot be equated with the number of unique individuals using a site. This is a well-recognized limitation of camera-trapping studies of unmarked species and is the reason we emphasize occupancy and detection-intensity interpretations over abundance or density.

## 5. Conclusions

This study provides the first occupancy-based analysis of environmental correlates of alpine musk deer occurrence in the central Qinghai–Tibet Plateau. Using systematic camera-trapping data from 221 stations and multi-framework analytical approaches, we found that elevation and slope are the dominant and most robust environmental correlates of site-level occupancy probability, whereas NDVI and distance-based proxy variables for vegetation productivity and human disturbance showed weak and unstable associations. Alpine musk deer activity was broadly cathemeral with a diurnal tendency, and significant variation in activity timing was detected amo.g months and between stations differing in proximity to roads and settlements, However, these patterns require confirmation with year-round data and direct measures of human activity.

Our results advance understanding of alpine musk deer ecology in three respects: (1) they establish quantitative occupancy–topography relationships for an understudied population; (2) they demonstrate the value of occupancy models for separating ecological signal from detection noise in musk deer research; and (3) they highlight the inferential limitations of remote-sensing proxy variables in complex mountain landscapes and provide a roadmap for incorporating direct field measurements in future work. We recommend that conservation planning in Baqing County and similar high-altitude pastoral landscapes prioritize the protection of steep, rugged terrain and valley-edge transition zones, and that future monitoring adopt repeated-detection designs capable of tracking occupancy trends, seasonal dynamics, and responses to changing land-use pressures.

## Figures and Tables

**Table 1:** Data sources.

Data	Source	Usage
Landsat 8/9 OLI	https://search.earthdata.nasa.gov/	NDVI calculation.
Land use	https://esri.maps.arcgis.com/	LandUse variable and extraction of water areas.
Google Earth high resolution imagery	https://earth.google.com/	Manual vectorization of roads and resident points
SRTM1 DEM (30 m)	https://search.earthdata.nasa.gov/	Elevation; derivation of slope and aspect

**Table 2:** Candidate occupancy model set.

Model	Occupancy (ψ) Formula	Detection (p) Formula	Hypothesis
Occu 1	1	effort + occasion	Null (constant occupancy)
Occu 2	Elevation + slope	effort + occasion	Topographic refuge
Occu 3	Elevation + slope + NDVI	effort + occasion	Topography + vegetation
Occu 4	Elevation + slope + NDVI + DisRoad + DisJM	effort + occasion	Full model (topography + vegetation + human proximity)
Occu 5	Elevation + slope + NDVI + DisWater + DisJM	effort + occasion	Water-source alternative

**Table 3:** Occupancy model selection results.

Model	K	AIC	ΔAIC	AIC Weight
Occu 2: ψ (Elevation + Slope) p (effort + occasion)	7	1562.3	0.00	0.62
Occu 3: ψ (Elevation + Slope + NDVI) p (effort + occasion)	8	1564.3	2.02	0.23
Occu 5: ψ (Elevation + Slope + NDVI + DisWater + DisJM) p (effort + occasion)	10	1567.1	4.82	0.06
Occu 4: ψ (Elevation + Slope + NDVI + DisRoad + DisJM) p (effort + occasion)	10	1567.4	5.14	0.05
Occu 1: ψ(.) p (effort + occasion)	5	1609.1	46.82	0.00

**Table 4:** Best-supported occupancy model (Occu 2) parameter estimates.

Component	Variable	β	SE	*p*	OR (95% CI)
ψ (occupancy)	Intercept	0.29	0.16	0.066	—
ψ (occupancy)	Elevation	−0.70	0.16	<0.001	0.50 (0.36–0.68)
ψ (occupancy)	Slope	0.58	0.17	<0.001	1.79 (1.27–2.51)
p (detection)	Intercept	−1.50	0.07	<0.001	—
p (detection)	Effort (log-days)	0.15	0.05	0.005	—

**Table 5:** Negative binomial model for alpine musk deer detection intensity.

Variable	IRR (95% CI)	*p* Value
Elevation	0.78 (0.60–1.03)	0.078
Slope	1.41 (1.08–1.86)	0.008
NDVI	1.18 (0.90–1.56)	0.200
DisRoad	0.86 (0.67–1.12)	0.260
DisJM	0.89 (0.72–1.11)	0.369

Note: IRR = incidence rate ratio for a one-SD increase. Model includes log (camera-trap days) as offset. Dispersion parameter (θ) = 0.32.

## Data Availability

The environmental data used in this study were obtained from publicly accessible platforms, namely Google Earth and the USGS EarthExplorer. Readers can access the raw data directly via these websites. The processed environmental covariate data are available from the corresponding author upon request. Exact camera-station coordinates are not publicly released because they could reveal sensitive wildlife-location information. The R analysis code used for data cleaning, descriptive statistics, regression modelling, count-model diagnostics, activity analysis, and figure generation can be provided upon reasonable request.

## Supplementary Materials

The following supporting information can be downloaded at: https://www.mdpi.com/article/10.3390/ani16162623/s1, Table S1. Logistic model AIC comparison table. Table S2. Missing-value summary. Table S3. Environmental characteristics of camera stations grouped by alpine musk deer detection status. Table S4. Summary of Alpine musk deer activity records. Table S5. Full logistic models. Table S6. Comparison of occupancy and logistic model effect estimates. Table S7. Count model comparison (Poisson, negative binomial, ZINB). Table S8. Spatial autocorrelation (Moran’s I) test results. Table S9. Monthly independent events, effective camera-days, and detection intensity. Table S10. Bootstrap validation of monthly and anthropogenic-proximity activity tests. Figure S1. Pearson correlation heatmap for continuous environmental variables. Figure S2. Exploratory temporal overlap between Alpine musk deer, red fox, and Tibetan fox. Figure S3. Boxplots of environmental gradients grouped by Alpine musk deer detection status. Boxplots illustrating the distribution of six environmental variables (Elevation, DisJM, DisRoad, DisWater, NDVI, and Slope) across non-detected (grey) and detected (orange) sites. Within each box, the horizontal line represents the median, the box bounds the 25th and 75th percentiles, the whiskers represent the data range within 1.5× the interquartile range, and the dots indicate outlier values. The results reveal that the detected sites were at significantly lower elevations, on steeper slopes, and closer to roads and water; NDVI and distance to settlements did not differ significantly. Figure S4. Predicted occupancy probability (ψ) as a function of elevation (DEM, m) and slope (degrees) derived from the top-ranked model. Shaded areas or lines represent predictions under different slope classes. Occupancy probability decreased sharply with increasing elevation, indicating a strong elevational constraint on habitat suitability. Figure S5. Monthly distribution of independent Alpine musk deer events. Figure S6. Species’ diel activity kernel density curves derived from a human-disturbance proxy (near vs. far from roads/settlements). Shaded regions or lines indicate estimated daily activity intensity for each disturbance category.
