# Supplementary material for: Fine-Scale Habitat Associations and Diel Activity Patterns of Camera-Detected Alpine Musk Deer (Moschus chrysogaster) in a High-Altitude Tibetan Landscape
Source: Animals (Basel). 2026 Aug 21;16(16):2623. doi: 10.3390/ani16162623 (PMC13509793; doi:10.3390/ani16162623)
Supplement: Supplementary file 1 [file animals-16-02623-s001.zip › animals-4467945-supplementary.pdf]

Supplementary Materials

Tables

Table S1. Logistic model AIC comparison table.

Model	Formula	AIC	ΔAIC	AIC weight
Logistic 1: topography vegetation effort	Musk detected = Elevation + Slope + NDVI + log trap days	277.98	0.00	0.64
Logistic 3: water settlement effort	Musk detected = Elevation + Slope + NDVI + DisWater + DisJM + log trap days	280.11	2.13	0.22
Logistic 2: road settlement effort	Musk detected = Elevation + Slope + NDVI + DisRoad + DisJM + log trap days	281.55	3.57	0.11
Logistic 4: aspect components sensitivity	Musk detected = Elevation + Slope + NDVI + DisRoad + DisJM + Aspect northness + Aspect eastness + log trap days	284.05	6.07	0.03

Table S2. Missing-value summary.

Variable	Missing	Missing (%)	Variable	Missing	Missing (%)
Longitude	0	0	Slope	0	0
Latitude	0	0	Aspect	0	0
Camera ID	0	0	DisWater	0	0
Elevation	0	0	Camera installation date	0	0
LandUse	0	0	Camera retrieval date	0	0
NDVI	0	0	Total camera-trap days	0	0
DisRoad	0	0	Aspect_rad	0	0
DisJM	0	0	Aspect_northness	0	0

Table S3. Environmental characteristics of camera stations grouped by alpine musk deer detection status.

Variable	Not detected (n = 98)	Detected (n = 123)	P value
Elevation (m)	4599 (4457–4759)	4470 (4357–4593)	< 0.001
Slope (°)	15.6 (8.7–25.4)	22.1 (14.2–30.2)	0.001
NDVI	0.34 (0.28–0.40)	0.34 (0.28–0.39)	1.000
DisRoad (m)	742 (315–1861)	360 (114–1178)	0.009
DisJM (m)	13322 (8251–19763)	15019 (7591–22675)	0.717
DisWater (m)	1437 (750–3065)	700 (292–3331)	0.030
Camera-trap days	93 (79–96)	92 (73–95)	0.207

Note: Values are medians (interquartile ranges). P values from Wilcoxon rank-sum tests.

Table S4. Summary of Alpine musk deer activity records.

Metric	Value
Independent events (30-min threshold)	1004
First detection time	2023-08-15 02:33:35
Last detection time	2023-11-25 06:02:52
Mean hour	12.36
Median hour	11.81
Rayleigh test statistic	0.082
Rayleigh test P value	0.001

Table S5. Full logistic models.

Model	Term	Estimate logit	Std error	Statistic	P. value	OR	95% CI
Occu 1	(Intercept)	0.25	0.15	1.67	0.10	1.28	0.96-1.71
Occu 1	Elevation	-0.70	0.16	-4.35	0.00	0.50	0.36-0.68
Occu 1	Slope	0.58	0.16	3.62	0.00	1.79	1.31-2.46
Occu 1	NDVI	-0.05	0.15	-0.30	0.76	0.96	0.71-1.29
Occu 1	Log trap days	-0.14	0.15	-0.93	0.35	0.87	0.65-1.17
Occu 2	(Intercept)	0.25	0.15	1.69	0.09	1.28	0.96-1.72
Occu 2	Elevation	-0.73	0.17	-4.30	0.00	0.48	0.35-0.67
Occu 2	Slope	0.60	0.17	3.60	0.00	1.82	1.31-2.52
Occu 2	NDVI	-0.03	0.15	-0.19	0.85	0.97	0.72-1.31
Occu 2	DisRoad	0.08	0.17	0.46	0.65	1.08	0.78-1.49
Occu 2	DisJM	-0.07	0.15	-0.45	0.66	0.93	0.69-1.26
Occu 2	Log trap days	-0.14	0.15	-0.95	0.34	0.87	0.65-1.16
Occu 3	(Intercept)	0.25	0.15	1.70	0.09	1.29	0.96-1.72
Occu 3	Elevation	-0.77	0.17	-4.52	0.00	0.46	0.33-0.65
Occu 3	Slope	0.60	0.17	3.57	0.00	1.82	1.31-2.52
Occu 3	NDVI	-0.02	0.15	-0.13	0.89	0.98	0.72-1.33

Model	Term	Estimate logit	Std error	Statistic	P. value	OR	95% CI
Occu 3	DisWater	0.21	0.17	1.26	0.21	1.23	0.89-1.70
Occu 3	DisJM	-0.07	0.15	-0.42	0.67	0.94	0.69-1.27
Occu 3	Log trap days	-0.14	0.15	-0.94	0.35	0.87	0.65-1.17
Occu 4	(Intercept)	0.26	0.15	1.75	0.08	1.30	0.97-1.73
Occu 4	Elevation	-0.72	0.17	-4.15	0.00	0.49	0.35-0.68
Occu 4	Slope	0.66	0.18	3.71	0.00	1.94	1.37-2.74
Occu 4	NDVI	0.00	0.16	-0.01	0.99	1.00	0.73-1.36
Occu 4	DisRoad	0.10	0.17	0.57	0.57	1.10	0.79-1.54
Occu 4	DisJM	-0.06	0.16	-0.41	0.68	0.94	0.69-1.27
Occu 4	Aspect northness	-0.18	0.16	-1.18	0.24	0.83	0.61-1.13
Occu 4	Aspect eastness	0.05	0.16	0.29	0.77	1.05	0.77-1.43
Occu 4	Log trap days	-0.14	0.15	-0.95	0.34	0.87	0.65-1.16

Note: Occu 1 is the topography-vegetation-effort model; Occu 2 is the road-settlement-effort model; Occu 3 is the water-settlement-effort model; Occu 4 includes aspect components. Elevation, Slope, NDVI, DisWater, DisJM, DisRoad, and log-transformed trap days were standardized.

Table S6. Comparison of occupancy and logistic model effect estimates.

Framework	Term	Estimate logit	Std error	P. value	OR	95% CI
Logistic detection/non-detection	Elevation	-0.73	0.17	0.00	0.48	0.35-0.67
Logistic detection/non-detection	Slope	0.60	0.17	0.00	1.82	1.31-2.52
Single-season occupancy	Elevation	-0.71	0.16	0.00	0.49	0.36-0.67
Single-season occupancy	Slope	0.58	0.16	0.00	1.79	1.27-2.51

Table S7. Count model comparison (Poisson, negative binomial, ZINB).

Model	AIC	ΔAIC	AIC weight	Dispersion or theta
Negative binomial GLM	1038.95	0.00	0.73	0.32
Zero-inflated negative binomial	1040.92	1.97	0.27	0.35
Poisson GLM	2486.92	1447.98	0.00	14.07

Table S8. Spatial autocorrelation (Moran's I) test results.

Model	Moran's I	Expectation	Variance	Statistic	P.value	k
Logistic Model 2 Pearson residuals	0.12	0.00	0.00	2.85	0.00	4
Logistic Model 2 Pearson residuals	0.15	0.00	0.00	4.52	0.00	6
Logistic Model 2 Pearson residuals	0.12	0.00	0.00	4.25	0.00	8
Negative binomial count model Pearson residuals	0.04	0.00	0.00	1.10	0.14	4
Negative binomial count model Pearson residuals	0.06	0.00	0.00	2.01	0.02	6
Negative binomial count model Pearson residuals	0.04	0.00	0.00	1.42	0.08	8

Table S9. Monthly independent events, effective camera-days, and detection intensity.

Month label	Independent events 30min	Effective camera-days	Events per 100 camera-days
2023-08	208	3,543	5.87
2023-09	338	6,359	5.32
2023-10	291	5,724	5.08
2023-11	167	2,664	6.27

Table S10. Bootstrap validation of monthly and anthropogenic-proximity activity tests.

Comparison	n	P min	P median	P max
Human-proximity proxy: near vs far	200	0.000	0.000	0.000
Monthly global: Aug-Sep-Oct-Nov	200	0.056	0.056	0.056
Monthly pairwise: 2023-08 vs 2023-09	200	0.420	0.420	0.420
Monthly pairwise: 2023-08 vs 2023-10	200	0.929	0.929	0.929
Monthly pairwise: 2023-08 vs 2023-11	200	0.004	0.004	0.004
Monthly pairwise: 2023-09 vs 2023-10	200	0.418	0.419	0.420
Monthly pairwise: 2023-09 vs 2023-11	200	0.106	0.106	0.106
Monthly pairwise: 2023-10 vs 2023-11	200	0.011	0.011	0.011

Figures

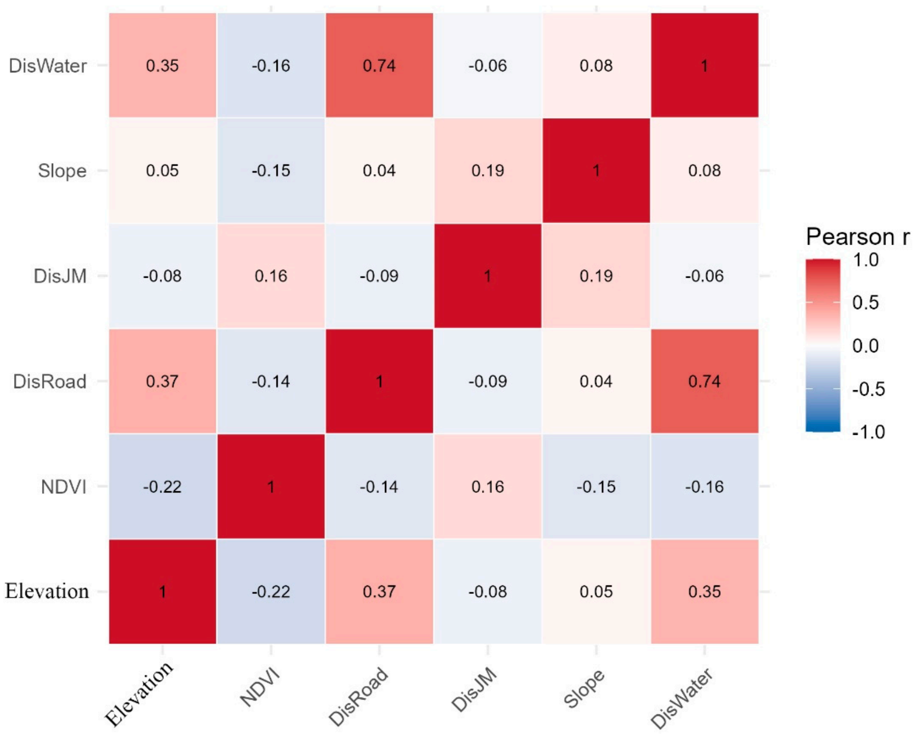

Figure S1. Pearson correlation heatmap for continuous environmental variables.

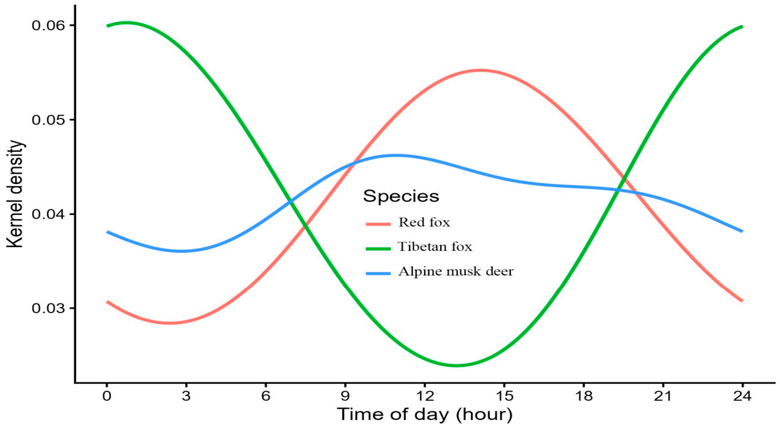

Figure S2. Exploratory temporal overlap between Alpine musk deer, red fox, and Tibetan fox.

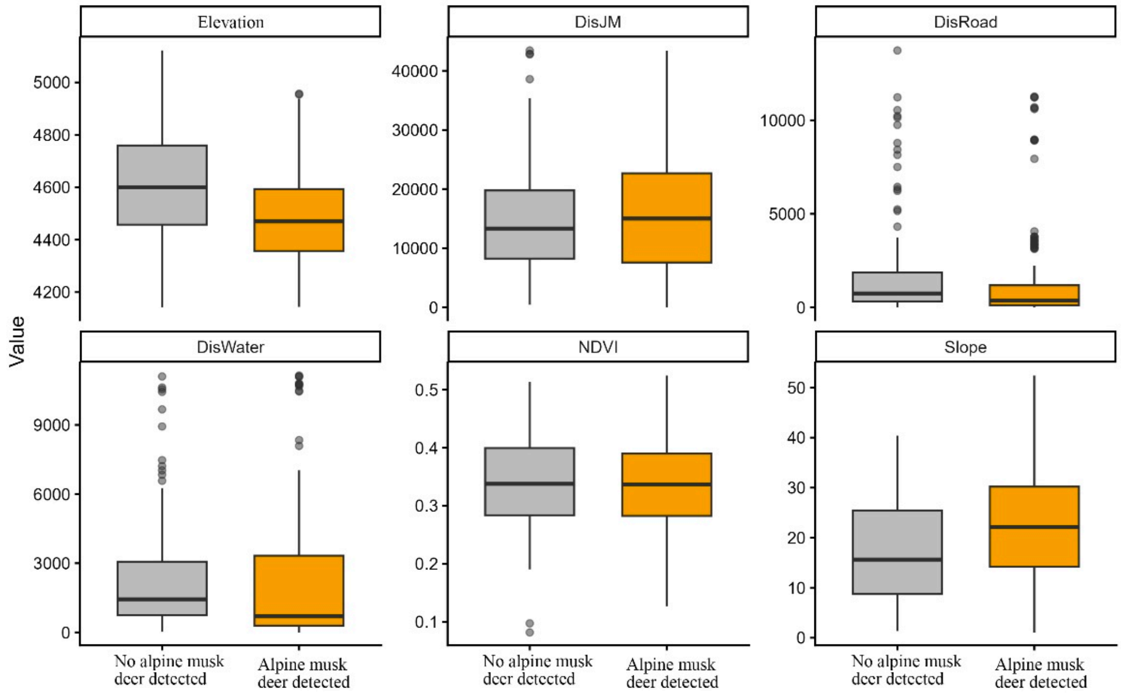

Figure S3. Boxplots of environmental gradients grouped by Alpine musk deer detection status. Boxplots illustrating the distribution of six environmental variables (Elevation, DisJM, DisRoad, DisWater, NDVI, and Slope) across non-detected (grey) and detected (orange) sites. Within each box, the horizontal line represents the median, the box bounds the 25th and 75th percentiles, the whiskers represent the data range within 1.5× the interquartile range, and the dots indicate outlier values. The results reveal that the detected sites were at significantly lower elevations, on steeper slopes, and closer to roads and water; NDVI and distance to settlements did not differ significantly.

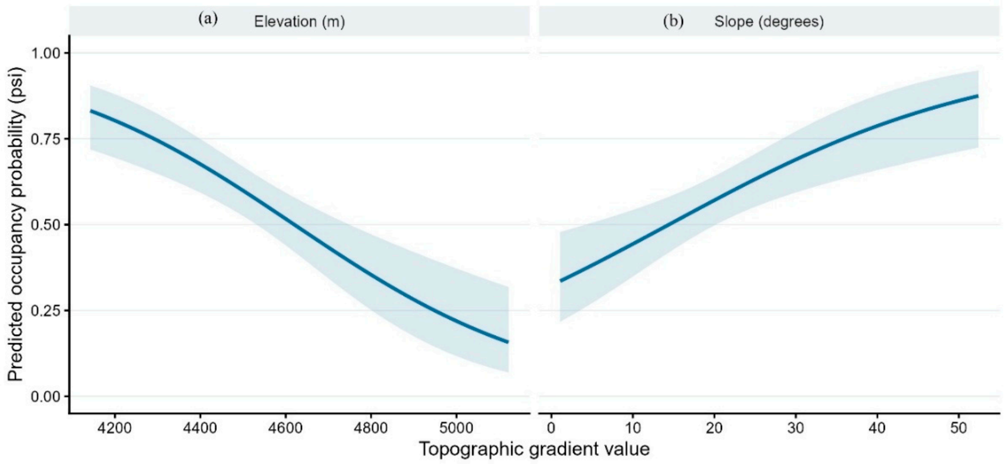

Figure S4. Predicted occupancy probability (ψ) as a function of elevation (DEM, m) and slope (degrees) derived from the top-ranked model. Shaded areas or lines represent predictions under different slope classes. Occupancy probability decreased sharply with increasing elevation, indicating a strong elevational constraint on habitat suitability.

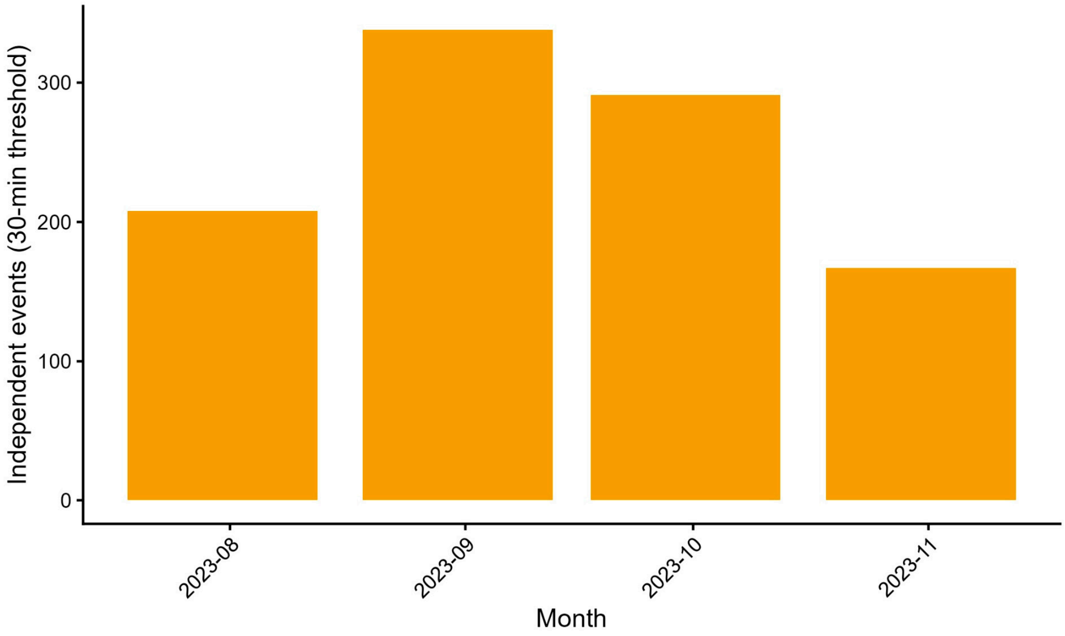

Figure S5. Monthly distribution of independent Alpine musk deer events.

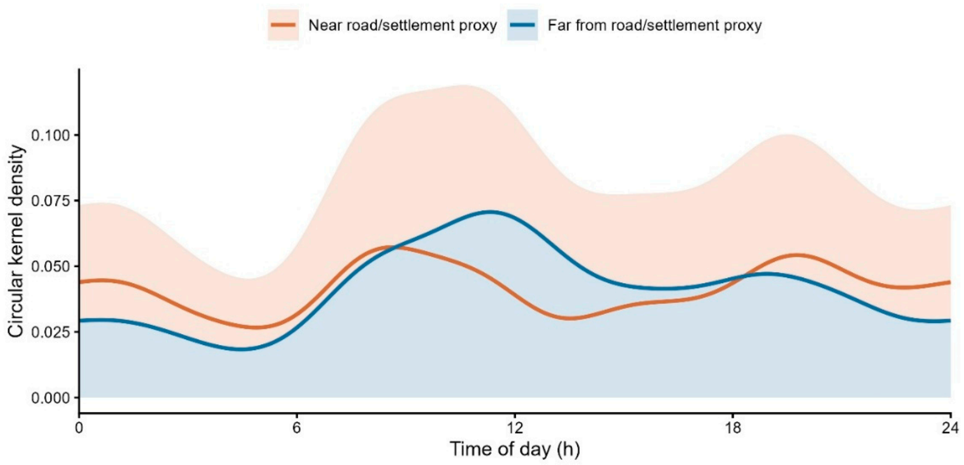

Figure S6. Species' diel activity kernel density curves derived from a human-disturbance proxy (near vs. far from roads/settlements). Shaded regions or lines indicate estimated daily activity intensity for each disturbance category.
